# Supplementary material for: The Portuguese version of the self-report form of the DSM-5 Level of Personality Functioning Scale (LPFS-SR) in a community and clinical sample
Source: PLoS One. 2024 Jun 27;19(6):e0300706. doi: 10.1371/journal.pone.0300706 (PMC11210752; doi:10.1371/journal.pone.0300706)
Supplement: S1 File — (PDF) [file pone.0300706.s001.pdf]

## Escala de Nível de Funcionamento da Personalidade – Autorrelato (LPFS-SR)

**Instruções:** Por favor, indique em que medida as afirmações que se seguem são verdadeiras para si:

**Chave de resposta:**

1 - Totalmente Falso, de maneira nenhuma Verdade

2 - Um pouco Verdade

3 - Maioritariamente Verdade

4 - Muito Verdade

| | | Totalmente Falso, de maneira nenhuma Verdade | Um pouco Verdade | Maioritariamente Verdade | Muito Verdade |
|---|---|---|---|---|---|
| 1 | Em muitas situações diferentes, consigo comportar-me de uma maneira adequada a cada situação. | 1 | 2 | 3 | 4 |
| 2 | Tudo o que eu consigo realmente perceber sobre as outras pessoas são as suas fraquezas. | 1 | 2 | 3 | 4 |
| 3 | Quase nenhuma relação próxima termina bem. | 1 | 2 | 3 | 4 |
| 4 | Embora possa ter sentimentos diferentes em momentos diferentes, consigo lidar com todos eles bastante bem. | 1 | 2 | 3 | 4 |
| 5 | Embora tente, não consigo manter relações duradouras e bem-sucedidas. | 1 | 2 | 3 | 4 |
| 6 | Embora valorize relações próximas, por vezes as emoções fortes atrapalham. | 1 | 2 | 3 | 4 |
| 7 | O que me acontece na vida pode mesmo influenciar se me sinto bem ou mal comigo próprio(a). | 1 | 2 | 3 | 4 |
| 8 | A opinião dos outros tem um papel determinante para eu saber o que é importante para mim. | 1 | 2 | 3 | 4 |
| 9 | Aproximar-me dos outros tem pouco interesse para mim. | 1 | 2 | 3 | 4 |
| 10 | Aproximar-me dos outros só me deixa vulnerável e não vale o risco. | 1 | 2 | 3 | 4 |
| 11 | Consigo dar valor ao ponto de vista de outras pessoas mesmo quando discordo delas. | 1 | 2 | 3 | 4 |
| 12 | Só me consigo aproximar de alguém que me entenda muito bem. | 1 | 2 | 3 | 4 |
| 13 | Só me consigo aproximar de alguém que reconheça e responda às minhas necessidades. | 1 | 2 | 3 | 4 |
| 14 | Consigo recuar para avaliar objetivamente o modo como me estou a sentir em qualquer momento. | 1 | 2 | 3 | 4 |
| 15 | Nem sempre consigo saber a diferença entre o que é a minha opinião e o modo como as outras pessoas querem que eu pense. | 1 | 2 | 3 | 4 |

LPFS-SR
Morey, L. C. (2017). Development and initial evaluation of a self-report form of the DSM-5 Level of Personality Functioning Scale. *Psychological Assessment*, *29*, 1302-1308. doi: 10.1037/pas0000450
Versão portuguesa (2018) da autoria de Rute Pires, Bruno Gonçalves, Joana Henriques-Calado (Faculdade de Psicologia da Universidade de Lisboa) e Leslie C. Morey (Texas A&M University).

| | | | | | |
|---|---|---|---|---|---|
| 16 | Nem sequer consigo imaginar viver uma vida em que me sinta realizado(a). | 1 | 2 | 3 | 4 |
| 17 | Não aguento quando há diferenças acentuadas de opinião. | 1 | 2 | 3 | 4 |
| 18 | Não faço a menor ideia por que razão as outras pessoas fazem o que fazem. | 1 | 2 | 3 | 4 |
| 19 | Não tenho muitas interações positivas com outras pessoas. | 1 | 2 | 3 | 4 |
| 20 | Não presto muita atenção, nem me preocupo muito, com o efeito que tenho sobre as outras pessoas. | 1 | 2 | 3 | 4 |
| 21 | Não entendo mesmo o que motiva as outras pessoas. | 1 | 2 | 3 | 4 |
| 22 | Não perco tempo a pensar sobre as minhas experiências, sentimentos e ações. | 1 | 2 | 3 | 4 |
| 23 | Tenho uma forte necessidade de que outros me aprovem. | 1 | 2 | 3 | 4 |
| 24 | Tenho dificuldade em definir e atingir objetivos. | 1 | 2 | 3 | 4 |
| 25 | Tenho pouca compreensão de como me sinto ou do que faço. | 1 | 2 | 3 | 4 |
| 26 | Tenho muitas relações satisfatórias, tanto pessoalmente quanto no trabalho. | 1 | 2 | 3 | 4 |
| 27 | Tenho relações, mas não muitas que considere muito próximas. | 1 | 2 | 3 | 4 |
| 28 | Tenho alguma dificuldade em estabelecer objetivos. | 1 | 2 | 3 | 4 |
| 29 | Tenho problemas em decidir entre dois objetivos diferentes. | 1 | 2 | 3 | 4 |
| 30 | Ajo sobretudo no momento, em vez de me concentrar em objetivos de longo prazo. | 1 | 2 | 3 | 4 |
| 31 | Presto atenção aos outros principalmente quando eles me podem afetar de alguma maneira. | 1 | 2 | 3 | 4 |
| 32 | Presto atenção às pessoas principalmente em função do que elas podem fazer por mim ou para mim. | 1 | 2 | 3 | 4 |
| 33 | Nunca tenho muita esperança de que coisas boas me venham a acontecer. | 1 | 2 | 3 | 4 |
| 34 | Estabeleço exigências para mim próprio(a) que são muito difíceis de satisfazer. | 1 | 2 | 3 | 4 |
| 35 | Costumo sentir-me ou muito bem ou muito mal comigo mesmo(a). | 1 | 2 | 3 | 4 |
| 36 | Costumo deixar que sejam os outros a definir os meus objetivos, em vez de ser eu próprio(a) a defini-los. | 1 | 2 | 3 | 4 |
| 37 | Esforço-me para ser flexível e cooperante ao lidar com os outros. | 1 | 2 | 3 | 4 |
| 38 | Normalmente entendo os sentimentos das outras pessoas melhor do que elas próprias. | 1 | 2 | 3 | 4 |
| 39 | Cuido das minhas relações próximas, porque elas são importantes para mim. | 1 | 2 | 3 | 4 |
| 40 | Sei distinguir os meus valores dos valores que os outros gostariam que eu tivesse. | 1 | 2 | 3 | 4 |
| 41 | Não tenho facilidade em recuar e olhar objetivamente para a minha vida. | 1 | 2 | 3 | 4 |

LPFS-SR
Morey, L. C. (2017). Development and initial evaluation of a self-report form of the DSM-5 Level of Personality Functioning Scale. *Psychological Assessment*, *29*, 1302-1308. doi: 10.1037/pas0000450
Versão portuguesa (2018) da autoria de Rute Pires, Bruno Gonçalves, Joana Henriques-Calado (Faculdade de Psicologia da Universidade de Lisboa) e Leslie C. Morey (Texas A&M University).

| | | | | | |
|---|---|---|---|---|---|
| 42 | Não tenho a certeza sobre exatamente que exigências defini para mim próprio(a). | 1 | 2 | 3 | 4 |
| 43 | Apenas me interessam relações que me proporcionem algum conforto. | 1 | 2 | 3 | 4 |
| 44 | Estou muito consciente do impacto que tenho nas outras pessoas. | 1 | 2 | 3 | 4 |
| 45 | Numa relação próxima, é como se eu não pudesse viver sem a outra pessoa. | 1 | 2 | 3 | 4 |
| 46 | Em relações próximas, costumo ficar dividido(a) entre ter medo e ser "pegajoso(a)". | 1 | 2 | 3 | 4 |
| 47 | Em muitas situações, sinto-me diferente do que os outros parecem esperar que eu sinta. | 1 | 2 | 3 | 4 |
| 48 | Em tempos muito difíceis, às vezes perco de vista o que é importante para mim. | 1 | 2 | 3 | 4 |
| 49 | Interagir com outras pessoas geralmente deixa-me confuso(a). | 1 | 2 | 3 | 4 |
| 50 | Parece-me que a maioria das outras pessoas tem a vida mais organizada do que eu. | 1 | 2 | 3 | 4 |
| 51 | Tenho objetivos que são razoáveis, dadas as minhas capacidades. | 1 | 2 | 3 | 4 |
| 52 | Tive relações duradouras, mas nem sempre foram muito satisfatórias. | 1 | 2 | 3 | 4 |
| 53 | A vida é um lugar perigoso e sem muito significado. | 1 | 2 | 3 | 4 |
| 54 | Muitas pessoas à minha volta têm motivações muito destrutivas. | 1 | 2 | 3 | 4 |
| 55 | A maioria das coisas que faço são uma reação ao que os outros fazem. | 1 | 2 | 3 | 4 |
| 56 | As minhas emoções mudam rapidamente. | 1 | 2 | 3 | 4 |
| 57 | A minha vida é basicamente controlada pelas ações dos outros. | 1 | 2 | 3 | 4 |
| 58 | As minhas motivações são-me essencialmente impostas, em vez de serem uma escolha pessoal. | 1 | 2 | 3 | 4 |
| 59 | As minhas exigências pessoais mudam bastante consoante as circunstâncias. | 1 | 2 | 3 | 4 |
| 60 | As outras pessoas geralmente esperam demasiado de mim. | 1 | 2 | 3 | 4 |
| 61 | As pessoas acham que eu sou bastante bom/boa a ler os sentimentos e motivações dos outros na maioria das situações. | 1 | 2 | 3 | 4 |
| 62 | As pessoas acham que eu sou “destrutivo(a)”, mas isso tem frequentemente mais a ver com elas do que comigo. | 1 | 2 | 3 | 4 |
| 63 | As relações são essencialmente uma fonte de dor e sofrimento. | 1 | 2 | 3 | 4 |
| 64 | Por vezes a única coisa que me interessa são os meus objetivos. | 1 | 2 | 3 | 4 |
| 65 | Por vezes sou muito duro(a) comigo mesmo(a). | 1 | 2 | 3 | 4 |
| 66 | Por vezes sinto que algumas pessoas são exatamente como eu; outras vezes acho que não são nada parecidas comigo. | 1 | 2 | 3 | 4 |
| 67 | Por vezes não sou muito colaborador(a) porque as outras pessoas ficam aquém das | 1 | 2 | 3 | 4 |

| | | | | | |
|---|---|---|---|---|---|
| | minhas exigências. | | | | |
| 68 | Por vezes é fácil para mim não me aperceber do impacto que tenho nos outros. | 1 | 2 | 3 | 4 |
| 69 | A chave para uma relação bem-sucedida é as minhas necessidades serem atendidas. | 1 | 2 | 3 | 4 |
| 70 | As exigências que coloco a mim próprio(a) ora são excessivas, ora não são suficientemente exigentes. | 1 | 2 | 3 | 4 |
| 71 | A maneira como os outros me veem é totalmente diferente da maneira como eu realmente sou. | 1 | 2 | 3 | 4 |
| 72 | Há partes da minha personalidade que não encaixam muito bem. | 1 | 2 | 3 | 4 |
| 73 | Ao lidar com pessoas, presto principalmente atenção ao modo como elas me podem afetar. | 1 | 2 | 3 | 4 |
| 74 | Quando os sentimentos ficam muito fortes, eu tento desligar-me deles. | 1 | 2 | 3 | 4 |
| 75 | Quando discordo dos outros, em geral não serve de nada tentar ver as coisas da sua perspetiva. | 1 | 2 | 3 | 4 |
| 76 | Quando sinto que fiz alguma coisa bem, quase sempre estou certo(a). | 1 | 2 | 3 | 4 |
| 77 | Quando não estou bem nalguma coisa, posso ficar com muita raiva ou sentir vergonha das minhas capacidades. | 1 | 2 | 3 | 4 |
| 78 | Quando tenho sucesso, tenho tendência para me sentir um(a) impostor(a). | 1 | 2 | 3 | 4 |
| 79 | Quando os outros me desaprovam tenho dificuldade em controlar as minhas emoções. | 1 | 2 | 3 | 4 |
| 80 | Quando penso sobre mim mesmo(a) posso ter uma visão muito limitada. | 1 | 2 | 3 | 4 |


Morey, L. C. (2017). Development and initial evaluation of a self-report form of the DSM-5 Level of Personality Functioning Scale. *Psychological Assessment*, *29*, 1302-1308. doi: 10.1037/pas0000450
Versão portuguesa (2018) da autoria de Rute Pires, Bruno Gonçalves, Joana Henriques-Calado (Faculdade de Psicologia da Universidade de Lisboa) e Leslie C. Morey (Texas A&M University).
