# Supplementary material for: The Portuguese version of the self-report form of the DSM-5 Level of Personality Functioning Scale (LPFS-SR) in a community and clinical sample
Source: PLoS One. 2024 Jun 27;19(6):e0300706. doi: 10.1371/journal.pone.0300706 (PMC11210752; doi:10.1371/journal.pone.0300706)
Supplement: S2 File — (PDF) [file pone.0300706.s002.pdf]

LPFS - SR

Name/ID: ___________________________

**Instructions:** For the following questions, please indicate the extent to which the statements are true for you:					
		**Totally False, not at all True**	**Slightly True**	**Mainly True**	**Very True**
1	Across many different situations, I manage to behave in a manner appropriate to that situation.	1	2	3	4
2	All I can really understand about other people are their weaknesses.	1	2	3	4
3	Almost no close relationship turns out well in the end.	1	2	3	4
4	Although I might have different feelings at different times, I can handle all of them pretty well.	1	2	3	4
5	Although I try, I can't seem to keep any successful, lasting relationships.	1	2	3	4
6	Although I value close relationships, sometimes strong emotions get in the way.	1	2	3	4
7	Events in my life can really change whether or not I feel good about myself.	1	2	3	4
8	Feedback from others plays a big role in determining what is important to me.	1	2	3	4
9	Getting close to others has little appeal to me.	1	2	3	4
10	Getting close to others just leaves me vulnerable and isn't really worth the risk.	1	2	3	4
11	I can appreciate the viewpoint of other people even when I disagree with them.	1	2	3	4
12	I can only get close to somebody who understands me very well.	1	2	3	4
13	I can only get close to someone who can acknowledge and address my needs.	1	2	3	4
14	I can step back and objectively evaluate the way that I'm feeling at any given time.	1	2	3	4
15	I can't always tell the difference between what is my opinion, and what is the way other people want me to think.	1	2	3	4
16	I can't even imagine living a life that I would find satisfying.	1	2	3	4
17	I can't stand it when there are sharp differences of opinion.	1	2	3	4
18	I don't have a clue about why other people do what they do.	1	2	3	4
19	I don't have many positive interactions with other people.	1	2	3	4
20	I don't pay much attention to, or care very much about, the effect I have on other people.	1	2	3	4
21	I don't understand what motivates other people at all.	1	2	3	4
22	I don't waste time thinking about my experiences, feelings, and actions.	1	2	3	4
23	I have a strong need for others to approve of me.	1	2	3	4
24	I have difficult setting and completing goals.	1	2	3	4
25	I have little understanding of how I feel or what I do.	1	2	3	4
26	I have many satisfying relationships, both personally and on the job.	1	2	3	4
27	I have relationships, but not many that I consider to be very close.	1	2	3	4
28	I have some difficulty setting goals.	1	2	3	4
29	I have trouble deciding between different goals.	1	2	3	4
30	I mainly act in the moment, rather than focusing on long term goals.	1	2	3	4
31	I mainly pay attention to others to the extent that they are likely to impact me in some way.	1	2	3	4
32	I mainly pay attention to people based upon what they might do to me, or for me.	1	2	3	4
33	I never seem to have much hope that good things will happen to me.	1	2	3	4
34	I set personal standards for myself that are very difficult to satisfy.	1	2	3	4

Copyright © 2016 Leslie C. Morey

LPFS - SR

Name/ID: ____________________________

Instructions: For the following questions, please indicate the extent to which the statements are true for you:

		Totally False, not at all True	Slightly True	Mainly True	Very True
35	I tend to feel either really good or really bad about myself.	1	2	3	4
36	I tend to let others set my goals for me, rather than come up with them on my own.	1	2	3	4
37	I try hard to be flexible and cooperative when dealing with others.	1	2	3	4
38	I typically understand other peoples' feelings better than they do.	1	2	3	4
39	I work on my close relationships, because they are important to me.	1	2	3	4
40	I'm confident about the difference between my values and those that others might want me to have.	1	2	3	4
41	I'm no good at stepping back and looking objectively at my life.	1	2	3	4
42	I'm not sure exactly what standards I've set for myself.	1	2	3	4
43	I'm only interested in relationships that can provide me with some comfort.	1	2	3	4
44	I'm very aware of the impact I'm having on other people.	1	2	3	4
45	In a close relationship, it's as if I can't live without the other person.	1	2	3	4
46	In close relationships I tend to be torn between being afraid and being "clingy".	1	2	3	4
47	In many situations I feel quite differently than others seem to expect me to feel.	1	2	3	4
48	In very trying times, I sometimes lose sight of what is important to me.	1	2	3	4
49	Interacting with other people usually leaves me feeling confused.	1	2	3	4
50	It seems as if most other people have their life together more than I.	1	2	3	4
51	I've got goals that are reasonable given my abilities.	1	2	3	4
52	I've had lasting relationships, but they haven't always been very satisfying.	1	2	3	4
53	Life is a dangerous place without a lot of meaning to it.	1	2	3	4
54	Many people around me have very destructive motives.	1	2	3	4
55	Most things that I do are a reaction to what others do.	1	2	3	4
56	My emotions rapidly shift around.	1	2	3	4
57	My life is basically controlled by the actions of others.	1	2	3	4
58	My motives are mainly imposed upon me, rather than being a personal choice.	1	2	3	4
59	My personal standards change quite a bit depending upon circumstances.	1	2	3	4
60	Other people often expect too much from me.	1	2	3	4
61	People think I am pretty good at reading the feelings and motives of others in most situations.	1	2	3	4
62	People think I'm a "hater", but it's often more related to them than to me.	1	2	3	4
63	Relationships are mainly a source of pain and suffering.	1	2	3	4
64	Sometimes all I care about are my goals.	1	2	3	4
65	Sometimes I am too harsh on myself.	1	2	3	4
66	Sometimes I feel that certain other people are just like me; other times I believe they are nothing like me at all.	1	2	3	4
67	Sometimes I'm not very cooperative because other people don't live up to my standards.	1	2	3	4
68	Sometimes it is easy for me to overlook the impact that I'm having on others.	1	2	3	4

Copyright © 2016 Leslie C. Morey

LPFS - SR

Name/ID: ___________________________

**Instructions:** For the following questions, please indicate the extent to which the statements are true for you:					
		**Totally False, not at all True**	**Slightly True**	**Mainly True**	**Very True**
69	The key to a successful relationship is whether I am getting my needs met.	1	2	3	4
70	The standards that I set for myself often seem to be too demanding, or not demanding enough.	1	2	3	4
71	The way that others perceive me is totally different from the way that I really am.	1	2	3	4
72	There are parts of my personality that just don't fit together very well.	1	2	3	4
73	When dealing with people, I mainly pay attention to how they are likely to affect me.	1	2	3	4
74	When feelings get too strong, I try to shut myself off from them.	1	2	3	4
75	When I disagree with others, there often isn't much use in trying to see things from their perspective.	1	2	3	4
76	When I feel that I've done something well, I'm almost always right.	1	2	3	4
77	When I'm not doing well at something, I might get very angry or feel ashamed about my abilities.	1	2	3	4
78	When I'm successful I tend to feel like an imposter.	1	2	3	4
79	When others disapprove of me, it's difficult to keep my emotions under control.	1	2	3	4
80	When thinking about myself, I can get pretty narrow in my focus.	1	2	3	4

Copyright © 2016 Leslie C. Morey

Subcomponent and Total Scoring: The Level of Personality Functioning Scale-Self Report (LPFS-SR)

Step 1: For each item, multiply the raw item score by the weight in the far-right column of the table below, and place the resulting product in the appropriate column in the table.

Step 2: Compute the subcomponent scores for Identity, Self-Direction, Empathy, and Intimacy by summing all of the weighted item scores in the corresponding column.

Step 3: Compute the Total Score by summing the four subcomponent scores calculated in Step 2.

Item	Identity	Self-Direction	Empathy	Intimacy	Weight
1					-0.5
2					2.5
3					2.5
4					-0.5
5					2.5
6					0.5
7					2.5
8					1.5
9					3.5
10					3.5
11					-0.5
12					1.5
13					1.5
14					-0.5
15					3.5
16					3.5
17					2.5
18					2.5
19					3.5
20					1.5
21					3.5
22					3.5
23					1.5
24					2.5
25					2.5
26					-0.5
27					1.5
28					0.5
29					0.5
30					3.5
31					1.5
32					3.5
33					2.5
34					0.5
35					2.5
36					1.5
37					-0.5
38					1.5
39					-0.5
40					-0.5
41					1.5
42					2.5
43					3.5
44					-0.5
45					2.5

46					2.5
47					3.5
48					0.5
49					3.5
50					1.5
51					-0.5
52					0.5
53					2.5
54					2.5
55					2.5
56					2.5
57					3.5
58					3.5
59					3.5
60					0.5
61					-0.5
62					3.5
63					3.5
64					0.5
65					0.5
66					2.5
67					0.5
68					0.5
69					3.5
70					1.5
71					3.5
72					2.5
73					2.5
74					0.5
75					0.5
76					-0.5
77					1.5
78					1.5
79					1.5
80					0.5
**TOTAL**					

Instructions to Clinicians

This Level of Personality Functioning Scale-Self Report (LPFS-SR) is an 80 item self-rated personality functioning assessment scale for adults age 18 and older. It assesses four interrelated core functions of personality, including Identity, Self-Direction, Empathy, and Intimacy, with each of these subcomponent scales consisting of 16 to 23 items. The four subcomponent scores are summed to yield an index of the level of severity of impairment in general personality functioning. The measure is completed by the individual prior to a visit with the clinician. Each item asks the individual to rate how well the item describes him or her generally.

Scoring and Interpretation

Each item on the measure is rated on a 4-point scale. The response categories for the items are 1=totally false, not at all true; 2=slightly true; 3=mainly true; 4=very true. None of the items are reverse-coded, although some are weighted negatively and thus deduct from score totals (see instructions above).

The weighted item scores are summed to form four subcomposites and a total score. These summary scores allow the clinician to think of the individual's severity of personality dysfunction relative to observed norms. Norms for the LPFS-SR are an ongoing process, but the scores below indicate various markers of elevated scores referenced against a sample of 306 non-clinical respondents. As scores exceed +1.0 standard deviation (SD) above the mean, subclinical problems may be expressed; scores exceeding 1.5 to 2.0 standard deviations above the mean may indicate clinically significant personality dysfunction that merits further assessment, treatment, and follow-up. Your clinical judgment should guide your decision.

	**Total Score**	**Identity**	**Self-Direction**	**Empathy**	**Intimacy**
Average	232.4	75.8	53.4	39.1	64.1
+ 1.0 SD	308.8	101.0	73.6	53.3	87.1
+ 1.5 SD	347.1	113.6	83.7	60.5	98.6
+ 2.0 SD	385.3	126.2	93.7	67.6	110.1

Notice: The LPFS-SR may be freely used, copied and redistributed without permission, but should be appropriately cited when used:

Morey, L.C. (2017). Development and initial evaluation of a self-report form of the DSM-5 Level of Personality Functioning Scale. *Psychological Assessment, 29*, 1302-1308. DOI: 10.1037/pas0000450
