# Supplementary material for: The Portuguese version of the self-report form of the DSM-5 Level of Personality Functioning Scale (LPFS-SR) in a community and clinical sample
Source: PLoS One. 2024 Jun 27;19(6):e0300706. doi: 10.1371/journal.pone.0300706 (PMC11210752; doi:10.1371/journal.pone.0300706)
Supplement: Attachment — Submitted filename: Response to Reviewers_12-02-24.docx [file pone.0300706.s005.docx]

*The Portuguese version of the self-report form of the DSM-5 Level of Personality Functioning Scale (LPFS-SR) in a community and clinical sample* [PONE-D-23-22309R1]

**Response to Reviewers**

We would like to thank Reviewers 3, 4 and 5 for recognizing and appreciating the amendments made, as well as for encouraging us to continue improving the paper.

Reviewer 4

In the following lines we respond to Reviewer 4’s comments and suggestions. Our comments and inserted text are in blue, and follow the order in which they were raised.

1. What was the criteria of the authors to determine the LPFS-SR cutoff scores? For example, Youden index or Gini index?

ROC curve analyses were used to examine the capacity of the LPFS-SR total score in discriminating between the samples. Optimal cutoff scores were determined to maximize the sum of sensitivity and specificity, ensuring the fewest incorrect decisions under conditions of equal a priori probabilities.

The Youden index is calculated as Sensitivity + Specificity - 1, and the Gini coefficient is given by 2 × AUC − 1.

2. According to the cut-off scores of the scale, the authors can report the true positive rate (TPR) and the true negative rate (TNR).

In our manuscript, we reported the sensitivity (true positive rate) and the specificity (true negative rate).

3. What is the similarity between the extracted factors with international studies? I recommend reporting Tucker’s congruence coefficients.

Tucker’s congruence coefficient is an index of the similarity between factor results (Lorenzo-Seva et al., 2006). The most popular tool for such comparisons was initially proposed by Burt (1948) and later gained popularity as Tucker’s congruence coefficient (Tucker, 1951). Its values range between -1 and +1.

Tucker’s congruence coefficient is defined as $\varphi_{C}= \frac{\sum x_{i}y_{i}}{\sqrt{{\sum x_{i}^{2}\sum y_{i}^{2}}}}$where x_i_ and y_i_ are the loadings of variable *i* on factor *X* and *Y*, respectively, i=1, …, n.

As suggested, we determined the Tucker’s congruence coefficient between the factor results of the Portuguese community sample, the Portuguese clinical sample, and the item factor loadings of Bliton et al. (2022). Unfortunately, we couldn't find other international factor results to compute the Tucker coefficient. Please see lines 235-238 and 383-392.

- Burt, C. (1948). The factorial study of temperament traits. *British Journal of Psychology, Statistical Section*, *1*, 178-203.

- Lorenzo-Seva, U., & Ten Berge, J. M. (2006). Tucker's congruence coefficient as a meaningful index of factor similarity. *Methodology*, *2*(2), 57-64.

- Tucker, L. R. (1951). A method for synthesis of factor analysis studies. *Personnel Research Section Report No.984,* Washington D.C.: Department of the Army.

4. Although examining convergent validity was probably not one of the goals of the study, if data are available, reporting correlations between LPFS-SR and other dimensional measures of personality would provide readers with additional information.

Data examining the LPFS-SR convergent validity with other dimensional measures of personality is already published. Please see the following reference for more details:

- Pires, R., Henriques-Calado, J., Sousa Ferreira, A., Gama Marques, J., Ribeiro Moreira, A., Barata, B. C., Paulino, M., & Gonçalves, B. (2023). Bridging the ICD11 and the DSM-5 personality disorders classification systems: The role of the PID5BF + M. *Frontiers in Psychiatry*, *14*, 1004895. https://doi.org/ 10.3389/fpsyt.2023.1004895

Reviewer 5

1. My only additional suggestion is that the authors mention the limitations of the study is that the validation of the Portuguese version of the LPFS-SR is not yet complete. This is due to the absence of scale reliability tests. According to COSMIN, in addition to internal consistency, reliability is an umbrella clinimetric property that encompasses reliability (the ability of the instrument to detect similar responses in repeated applications on the same patient, if he/she is clinically stable) and the error of measurement (size of the error systematically present during the application of the instrument). Along with criterion and discriminant validity, reliability would ensure the complete accuracy of the scale for use in clinical practice and scientific research. However, the reliability test depends on a test and retest design, and this was probably the reason for its failure.

We have followed Reviewer 5's suggestion, which has been implemented in the following lines: 433-437.
